# The Benefits of Active Participation in the European Federation of Psychiatric Trainees (EFPT) for Psychiatric Trainees Across Europe

**DOI:** 10.1192/j.eurpsy.2025.2378

**Published:** 2025-08-26

**Authors:** F. M. Monshizadeh Tehrani, N. de Ridder, V. H. Santos, M. Konings

**Affiliations:** 1 European Federation of Psychiatric Trainees, Brussels, Belgium; 2 President, European Federation of Psychiatric Trainees, Brussels, Belgium

## Abstract

**Introduction:**

The European Federation of Psychiatric Trainees (EFPT) unites general adult psychiatry (GAP) and child and adolescent psychiatry (CAP) trainees’ associations across Europe, fostering collaboration, education, and professional growth. Trainees across Europe can become EFPT members through their national associations (NTA). Residency can be challenging, with demanding workloads and academic pressures. Having a supportive network like EFPT can ease these difficulties by providing access to valuable resources, support, and collaboration. This abstract highlights the benefits of active participation in EFPT for GAP and CAP trainees across Europe.

**Objectives:**

To explore the diverse benefits provided by EFPT for GAP and CAP trainees across Europe, focusing on professional development, research collaborations, and international exchanges. This abstract highlights strategies for maximizing these opportunities and overcoming the challenges of international psychiatric training.

**Methods:**

A review of EFPT’s initiatives, project descriptions, website data, and participant feedback was conducted. This analysis covered webinars, courses, research projects, clinical exchanges, and cultural experiences. Data were gathered from EFPT’s resources and European trainees’ feedbacks, emphasizing networking, research collaboration, and cultural exchange as key benefits for trainees.

**Results:**

EFPT offers a broad range of benefits for GAP and CAP traineesroughout Europe, including:

- **Networking Opportunities:** Trainees can establish professional connections with peers and experienced psychiatrists from across Europe, fostering collaboration, mentorship, and knowledge sharing.
- **International Participation:** Trainees have access to international congresses, webinars, and specialized courses that enhance clinical and research skills, exposing them to diverse perspectives and practices.
- **Clinical Exchange Programs:** Through EFPT’s exchange programs, trainees gain insights into different healthcare systems, broadening their understanding of child and adolescent mental health treatment across Europe and enhancing their cultural competence.
- **Cultural Enrichment:** EFPT’s annual forum, including events like Cultural Night, allows trainees to engage in cultural exchange through music, food, and dance, fostering camaraderie and promoting overall well-being among young psychiatrists.

**Conclusions:**

Active participation in EFPT offers European psychiatric trainees unique opportunities beyond traditional training, contributing to both professional growth and personal development. By encouraging involvement in EFPT, trainees can enhance their skills, broaden their networks, and foster international collaboration, ultimately strengthening the psychiatric community across Europe.

**Disclosure of Interest:**

None Declared

